# Lesion outline and thermal field distribution of ablative in vitro experiments in myocardia: comparison of radiofrequency and laser ablation

**DOI:** 10.1186/s12872-020-01735-3

**Published:** 2020-10-20

**Authors:** Wei Li, Jia Liu, Tong-yi Huang, Xian Zhong, Dao-peng Yang, Xiao-hua Xie, Dong-hong Liu, Xiao-yan Xie, Bo-wen Zhuang

**Affiliations:** 1 Department of Medical Ultrasonics, The First Affiliated Hospital of Sun Yat-Sen University, Guangzhou, 510080 Guangdong China; 2 Department of Medical Ultrasonics, Division of Interventional Ultrasound, Institute of Diagnostic and Interventional Ultrasound, The First Affiliated Hospital of Sun Yat-Sen University, Guangzhou, 510080 Guangdong China

**Keywords:** Radiofrequency ablation, Laser ablation, Thermal field distribution, Hypertrophic obstructive cardiomyopathy, In vitro

## Abstract

**Objectives:**

To explore the lesion outline and thermal field distribution of radiofrequency ablation (RFA) and laser ablation (LA) in myocardial ablation in vitro.

**Materials and methods:**

Twenty-four fresh porcine hearts were ablated with RFA or LA in vitro. The radiofrequency electrode or laser fiber and two parallel thermocouple probes were inserted into the myocardium under ultrasound guidance. The output power for RFA was 20 W/s and for LA was 5 W/s, and the total thermal energies were 1200 J, 2400 J, 3600 J, and 4800 J. The range of ablation lesions was measured, and temperature data were recorded simultaneously.

**Results:**

All coagulation zones were ellipsoidal with clear boundaries. The center of LA was carbonized more obviously than that of RFA. With the accumulation of thermal energy and the extended time, all the ablation lesions induced by both RFA and LA were enlarged. By comparing the increase in thermal energy between the two groups, both the short-axis diameter and the volume change showed significant differences between the 1200 J and 3600 J groups and between the 2400 J and 4800 J groups (all *P* < 0.05). Both the short-axis diameter and the volume of the coagulation necrosis zone formed by LA were always larger than those of RFA at the same accumulated thermal energy. The temperatures of the two thermocouple probes increased with each energy increment. At the same accumulated energy, the temperature of LA was much higher than that of RFA at the same point. The initial temperature increase at 0.5 cm of LA was rapid. The temperature reached 43 °C and the accumulated energy reached 1200 J after approximately 4 min. After that the temperature increased at a slower rate to 70  C. For the RFA at the point of 0.5 cm, the initial temperature increased rapidly to 30 °C with the same accumulated energy of 1200 J after only 1 min. In the range of 4800 J of accumulated thermal energy, only the temperature of LA at the point of 0.5 cm exceeded 60 °C when the energy reached approximately 3000 J.

**Conclusions:**

Both RFA and LA were shown to be reliable methods for myocardial ablation. The lesion outline and thermal field distribution of RFA and LA should be considered when performing thermal ablation in the intramyocardial septum during hypertrophic obstructive cardiomyopathy.

## Introduction

Over the past decade, invasive therapeutic options for hypertrophic obstructive cardiomyopathy (HOCM), including surgical myectomy, alcohol septal ablation (ASA), radiofrequency catheter ablation (RFCA), dual-chamber pacing and implantable cardioverter defibrillator (ICD), have been developed to improve clinical symptoms and to relieve left ventricular outflow tract (LVOT) obstruction [1–6]. Recently, a novel therapy of transthoracic echocardiography–guided percutaneous intramyocardial septal radiofrequency ablation (PIMSRA) has been investigated [7, 8]. PIMSRA is a percutaneous intramyocardial, non-transaortic and non-transcoronary operation to reduce the LVOT obstruction. This procedure could avoid traditional sternotomy and damage to the conduction system, which is distributed underneath the endocardium. The treatment can effectively improve the hemodynamics and symptom of patients with HOCM during 6 months of follow-up [7]. To protect the conduction system, the investigators recommended maintaining a 3 mm-safe margin between the outline of the ablated zone and the endocardium of both left and right ventricle. Although an ECG monitor is used to demonstrate the change in rhythm or configuration, it is meaningful to pre-estimate the range of coagulation necrosis ablation lesions.

Currently, this procedure is performed by ablating the hypertrophic interventricular septum (IVS) with a radiofrequency needle. Radiofrequency ablation (RFA) is a safe and effective treatment method for tumors, and it can obtain a definite and stable ablation boundary [9, 10]. Unlike tumor ablation, cardiac ablation is concerned with safety boundaries to avoid injuring tracts. In addition to RFA, laser ablation (LA) can also obtain a definite ablation boundary, and this procedure is usually used in small organs such as the thyroid and prostate [11, 12]. Moreover, the needle of LA is 21-gauge, which is much finer than the 17-gauge needle used for RFA. This difference may result in less injury and bleeding. Numerous trials with different treatment algorithms have confirmed the clinical effectiveness and safety of LA in solid thyroid nodules or lesions with variable fluid components [12–14]. Therefore, LA might be a potential option for cardiac ablation.

In this study, we used porcine hearts to investigate the lesion outline and thermal field distribution of RFA and LA for myocardial ablation in vitro according to different thermal energies and power outputs.

## Materials and methods

No institutional review board approval was necessary, as no human subjects participated in this study. Furthermore, animal committee approval was not necessary, as we used an in vitro porcine heart model. Twenty-four fresh room-temperature porcine hearts were offered by the local butcher at the same vendor. The average weight of the porcine hearts was 500 ± 50 g.

RFA was performed by using the Cool-tip™ radiofrequency system (Valleylab, Boulder, CO, USA). This system consists of the following components: an RF generator (maximum power: 200 W, electric current: 480 kHz), a 17-gauge internally cooled monopolar electrode with a 2-cm exposed tip, a peristaltic perfusion pump, and a 10-cm^2^ grounding pad. The porcine hearts were immersed in saline. The grounding pad was put at least 30 cm away from the electrode. A mechanical pump was used to cool the electrode with the internal circulation of sterile saline (4 °C), and the flow rate of the circulation was approximately 100 mL/min. During RFA, the electrode was placed 3 cm into the porcine heart. The output power was controlled at 20 W/s, with the ablation times were set as 1 min, 2 min, 3 min and 4 min.

The LA equipment was EchoLaser type X4 (Esaote Company, Florence, Italy) with a 300 μm plane-cut optic fiber sheathed by a 21-gauge PTC needle. It radiated laser light at a wavelength of 1064 nm. The PTC needle was inserted into the target position under the guidance of ultrasound, and then the core needle was pulled out. The fiber was inserted through the needle sheath into the same position. Then the PTC needle was withdrawn for 5 mm, leaving the tip of the fiber in direct insert into the tissue. LA were lunched with an output power of 5 W, and the gross energies were set at 1200 J, 2400 J, 3600 J and 4800 J.

**Fig. 1:**
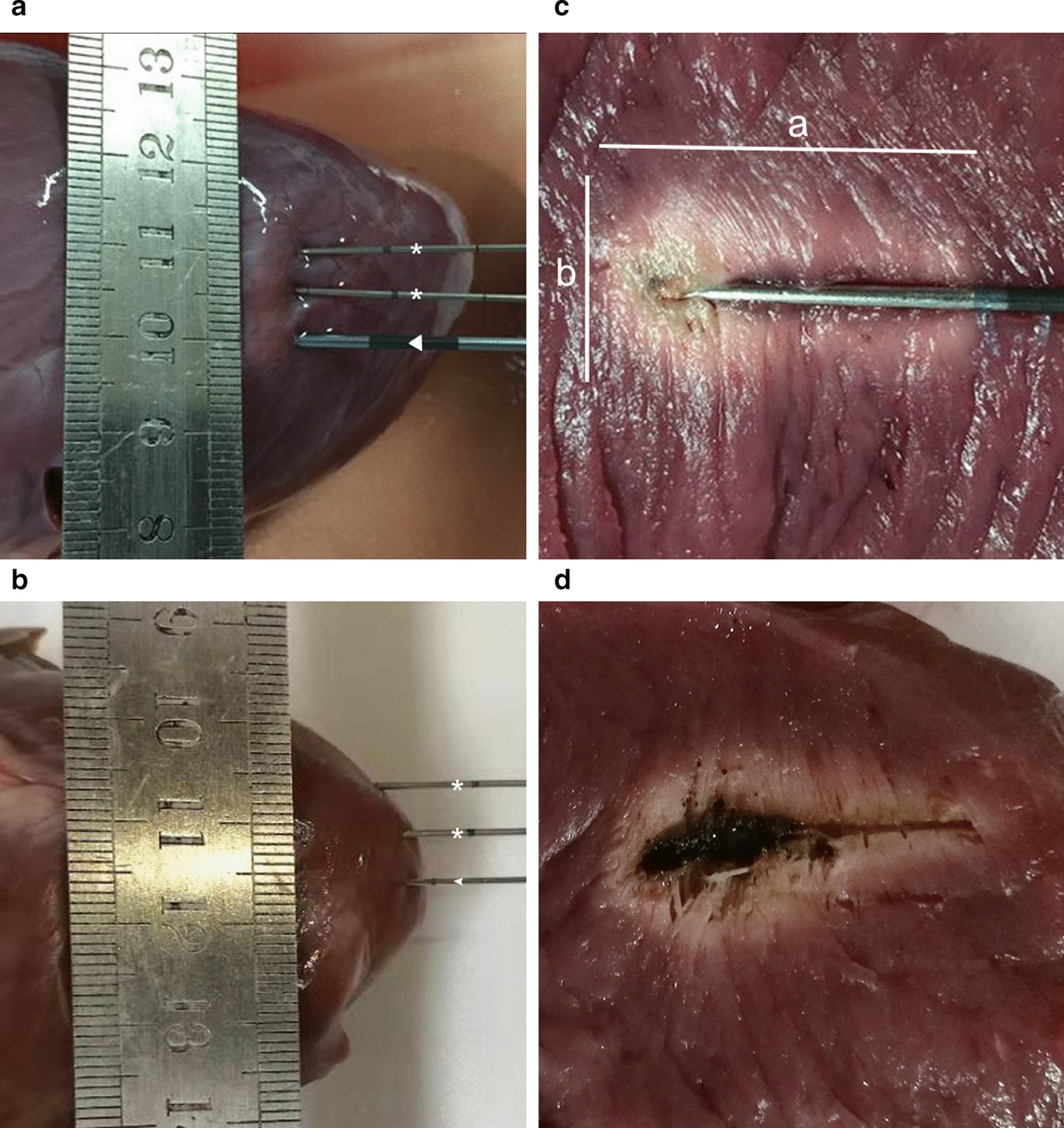
**a** Photograph shows radiofrequency ablation (triangle) in porcine heart. Two thermocouple probes (asterisk) were placed at the same depth parallel to the needle at distances of 5 mm and 10 mm to monitor the local tissue temperature during the procedure. **b** Photograph shows laser ablation (arrowhead) in porcine heart. Two thermocouple probes (asterisk) were placed at the same depth parallel to the needle at distances of 5 mm and 10 mm to monitor the local tissue temperature during the procedure. **c** The coagulation necrosis zones of radiofrequency ablation. The long-axis diameter (**a**) and short-axis diameter (**b**) are shown. **d** The coagulation necrosis zones of laser ablation. The center of laser ablation was carbonized more obviously than that of radiofrequency ablation

Before ablation, ultrasonography was used to guide the insertion of the electrode or laser fiber. The electrode or laser fiber was inserted into the thickest part (2–3 cm) of the interventricular septum of each porcine heart. Temperatures were measured throughout each ablation by using a SENDAE SD-TC02B thermal monitor (Sun-Gun Automation Engineer Company, Guangzhou, China). Two thermocouple probes were implanted at the same depth parallel to the needle at distances of 5 mm and 10 mm (Fig. 1a, b). Only the thermocouple tip could measure the temperature that the thermocouple tip was monitored by using real-time ultrasound imaging to guarantee its position. The temperature data were collected every 10 s for the entirety of each ablation.

Each post-ablation coagulation specimen was sectioned along the electrode shaft. The coagulative necrosis zone along the needle insertion axis was considered as the long-axis diameter, while the short-axis diameter was measured perpendicular to the long-axis diameter (Fig. 1c). According to previous experiments, the diameters of short-axis were usually symmetrical in this in vitro experiment. Thus, the short-axis diameter was used twice for the volume calculations. The coagulation volume (V) was calculated by using the formula for ellipsoids: V = (π × long-axis diameter × short-axis diameter × short-axis diameter)/6 [15]. Measurements were performed only on the central zone of coagulative necrosis, which consisted of a central charring zone and a white coagulation zone. Two individuals (WL, JL) working in consensus measured all ablation zone. All of the operations described above were performed three times in each group.

### Statistical analysis

Data analysis was performed with SPSS 22.0 software (Inc., Chicago, IL). The results are presented as the mean ± SEM (standard error of the mean). Comparisons between two groups were statistically analyzed using a two-sided Student’s t test, and statistical studies between multiple groups were analyzed using multiway analysis of variance (ANOVA). The temperature rise curves were plotted in Origin software (Origin Lab, version 8.5). The method of repeated measurement and variance analysis was used. All data with *P* values less than 0.05 indicated statistically significant differences.

## Results

The coagulation necrosis zones in the porcine hearts after ablation were pale and hard. The ablation zone was almost ellipsoidal with a clear boundary and did not include the undesired extension of coagulation along the needle shaft. The center of LA was carbonized more obviously than that of RFA (Fig. 1d).

### The comparison of lesion outlines between the two ablation modes

The size changes, including the long-axis diameter, short-axis diameter and volume, after thermal ablation under different thermal energies induced by LA and RFA are shown in Table 1. All the long-axis diameters, short-axis diameters and volumes of both thermal methods were enlarged with increasing thermal energy.

**Table 1:** The long-axis diameter, short-axis diameter and volume change of coagulations at different thermal energy by laser ablation and radiofrequency ablation

Energy (J)	LA			RFA		
	Long-axis diameter (cm)	Short-axis diameter (cm)	Volume (cm^3^)	Long-axis diameter (cm)	Short-axis diameter (cm)	Volume (cm^3^)
1200	2.1 ± 0.1	1.1 ± 0.1	1.3 ± 0.3	1.2 ± 0.1	0.6 ± 0.0	0.3 ± 0.0
2400	2.2 ± 0.1	1.5 ± 0.1	2.4 ± 0.5	2.2 ± 0.1	0.8 ± 0.1	0.8 ± 0.1
3600	2.4 ± 0.1	1.9 ± 0.1	4.2 ± 0.1	2.3 ± 0.1	1.1 ± 0.1	1.5 ± 0.1
4800	2.5 ± 0.1	2.1 ± 0.1	5.3 ± 0.2	2.3 ± 0.0	1.4 ± 0.1	2.2 ± 0.2

**Fig. 2:**
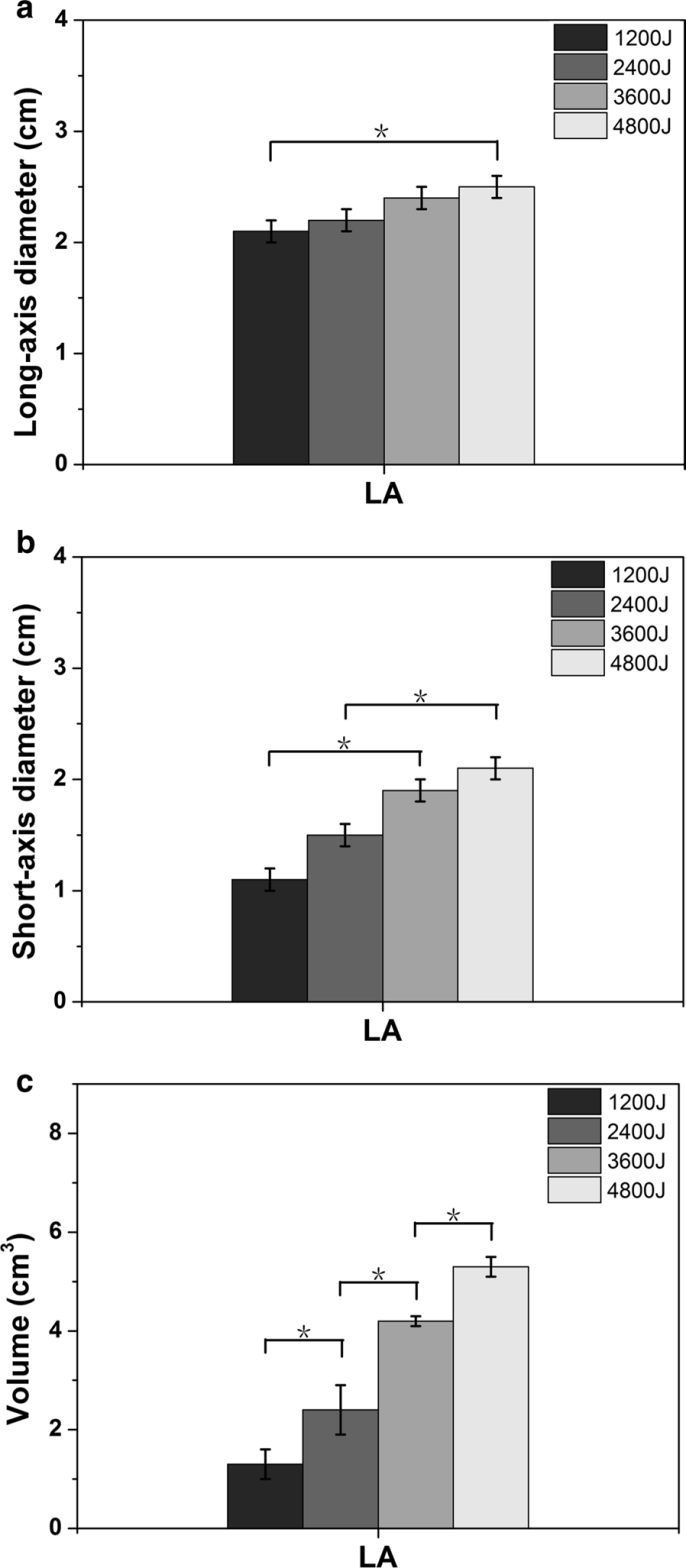
The changes in the **a** long-axis diameter, **b** short-axis diameter and **c** volume after myocardial ablation with different laser ablative energies. *The results between the different energies are significantly different (*P* < 0.05)

**Fig. 3:**
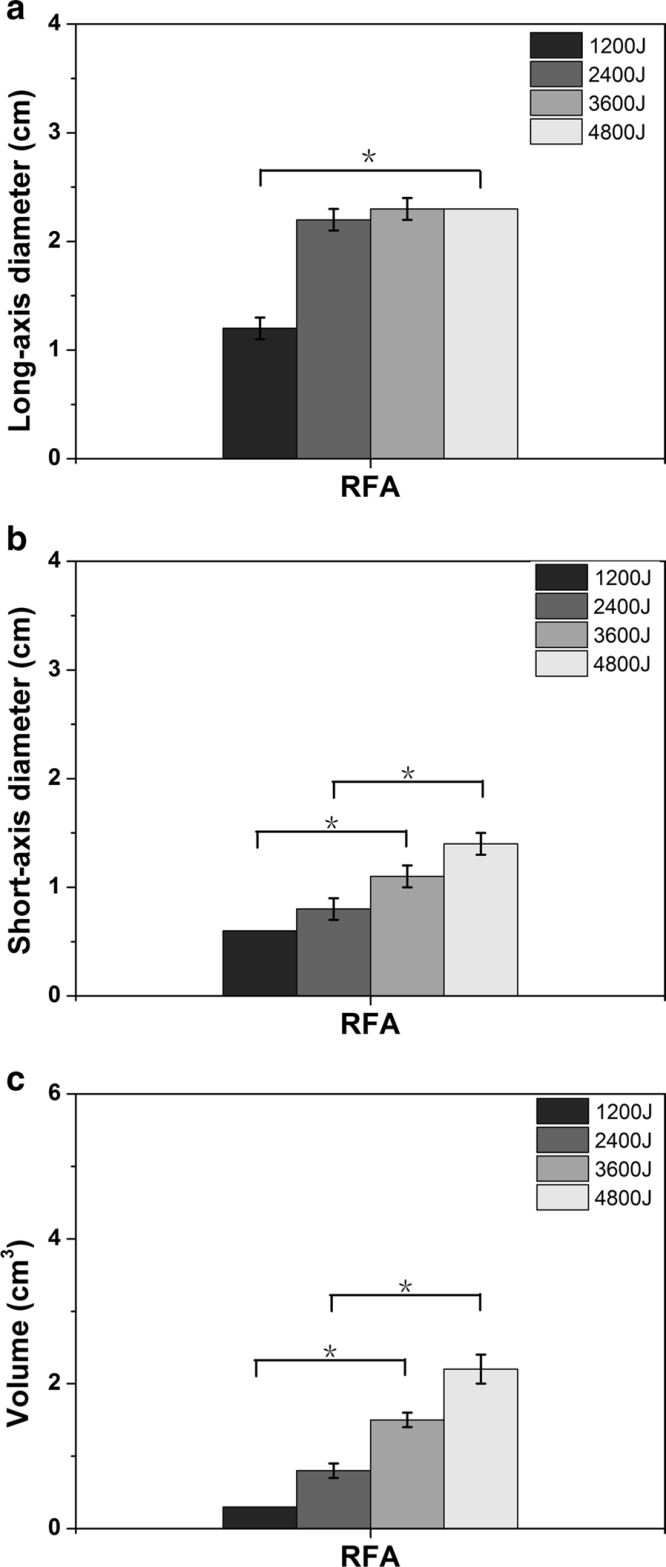
The changes in the **a** long-axis diameter, **b** short-axis diameter and **c** volume after myocardial ablation with different radiofrequency ablative energies. *The results between the different energies are significantly different (*P* < 0.05)

**Fig. 4:**
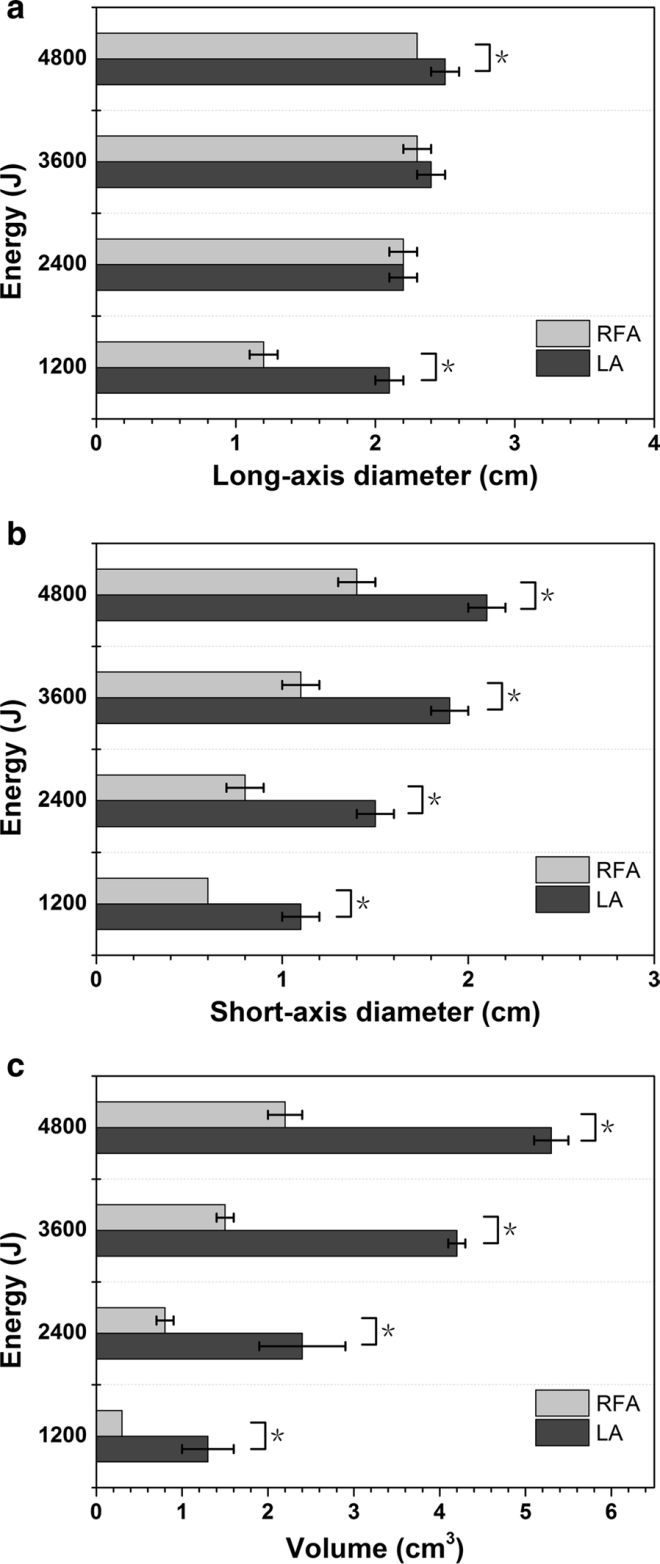
Graphs show the **a** long-axis diameter, **b** short-axis diameter and **c** volume of the coagulation zone with laser ablation and radiofrequency ablation at the same energy. Error bars = 95% confidence interval. *The results between the two ablation systems are significantly different (*P* < 0.05)

For LA, the smallest ablation lesion was 1.3 ± 0.3 cm^3^ in volume, with a long-axis diameter of 2.1 ± 0.1 cm and a short-axis diameter of 1.1 ± 0.1 cm at 1200 J, while the largest ablation lesion was 5.3 ± 0.2 cm^3^ in volume, with a long-axis diameter of 2.5 ± 0.1 cm and a short-axis diameter of 2.1 ± 0.1 cm at 4800 J. The long-axis diameter at 1200 J was 2.1 ± 0.1 cm, which was significantly different from that of the 4800 J group (2.5 ± 0.1 cm, *P* < 0.05). The short-axis diameter changed significantly between 1200 J (1.1 ± 0.1 cm) and 3600 J (1.9 ± 0.1 cm) and between 2400 J (1.5 ± 0.1 cm) and 4800 J (2.1 ± 0.1 cm) (all *P* < 0.05). The volume change was significantly different between all the groups (all *P* < 0.05) (Fig. 2). For RFA, the smallest ablation lesion was 0.3 ± 0.0 cm3 in volume, with a long-axis diameter of 1.2 ± 0.1 cm and a short-axis diameter of 0.6 ± 0.0 cm at 1200 J, while the largest ablation lesion was 2.2 ± 0.2 cm3 in volume, with a long-axis diameter of 2.3 ± 0.0 cm and a short-axis diameter of 1.4 ± 0.1 cm at 4800 J. By comparing the increases in thermal energy between the two groups, the long-axis diameter change was statistically significant only between 1200 and 4800 J; the short-axis diameter and the volume change showed significant differences between 1200 and 3600 J and between 2400 and 4800 J (Fig. 3). At the same accumulated thermal energy, LA had a larger long-axis diameter than RFA at 1200 J and 4800 J. Both the short-axis diameter and volume of the coagulation necrosis zone formed by LA were always larger than those of RFA at each accumulated thermal energy (Fig. 4).

### Temperature measurement evaluation

**Fig. 5:**
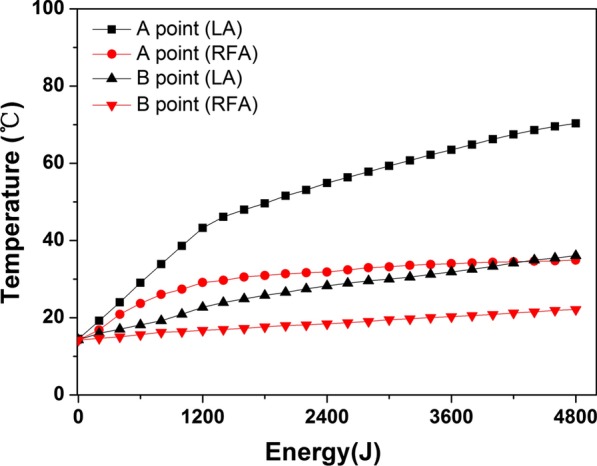
Graphs show the temperature curves of the thermal distribution at different energies with laser ablation (black curves) and radiofrequency ablation (red curves)

The average temperature measurements of the two thermocouple probes are shown in Fig. 5. The temperature increased with each energy increment. The temperature near the antenna at 0.5 cm showed a higher and greater change than that near the far-field region at 1.0 cm due to the localization of heating. In contrast, the temperature curves in the region far from the antenna were much smoother, since thermal conduction led to the rise in temperature. At the same accumulated energy, the temperature of LA was much higher than that of RFA at the same point. Interestingly, the initial temperature increase of LA at 0.5 cm was rapid. The temperature reached 43 °C at an accumulated energy of 1200 J after approximately 4 min. And after that, the temperature increased at a slower rate from 43 °C to 70 °C. For the RFA at the point of 0.5 cm, the initial temperature rose rapidly to 30 °C with the same accumulated energy of 1200 J after only 1 min, after which the temperature nearly plateaued. It is worth noting that in the range of 4800 J of accumulated thermal energy, only the temperature of LA at the point of 0.5 cm exceeded 60 °C when the energy reached approximately 3000 J.

## Discussion

In this study, with increasing accumulated thermal energy, both the long-axis diameter and short-axis diameter were enlarged, especially for the short-axis diameter of RFA that reached 14 mm and that of LA that reached 21 mm, which included the standard of ablation for ventricular septal thickness (≥ 15 mm). The size of the ablation lesions formed by LA was larger than that formed by RFA under the same output energy, showing a greater efficiency accomplished by LA than by RFA in our settings (< 4800 J). In terms of the thermal distribution, the temperature of the far-field region was lower and increased more slowly compared with that of the central zone for both LA and RFA. Moreover, the temperature of the LA group was always higher than that of the RFA group at the same point under the same output energy, which also suggested that LA might be more efficient than RFA under the same energy consumption.

Minimally invasive thermal ablation of lesions has become common since the advent of modern guidance method [16]. Percutaneous thermal ablation is initially used for the treatment of small, unresectable tumors or for patients who are poor surgical candidates [17–20]. As technology advances and more exploration, thermal ablation will no longer be confined to the treatment of tumors but will be used in other tissues and organs as the lung and heart. RFA is the most widely used ablation method, especially for the therapy of hepatocellular carcinoma (HCC) [21]. The heating principle of RFA is that the radiofrequency electrode generates an electric field with a high-frequency alternating current. Frictional heating was generated when the ions in the tissue attempt to follow the changing directions of the alternating current. LA is another minimally invasive local-ablative technique which is less investigated and used compared to RFA. However, some published data have shown that LA is equivalent to RFA in terms of both tumor control and long-term outcomes for the percutaneous treatment of HCC [22–24]. The principle of LA is based on the spontaneous emission of characteristic photons by excited atoms and light energy produced by laser equipment from electrical energy acting on tissues to generate heat. The local temperature could rise to above 200 °C, which cause the local tissue coagulated, became necrotic, charred, or even vaporized. However, because the light of laser is easily scattered and absorbed, this modality has limited tissue penetration and hence the ablation areas is very small of approximately 1–2 cm^2^. Under these conditions, LA is typically used in the field of small organs such as thyroid, prostate and nerves [25–29]. Therefore, LA has advantages in terms of laser precision and efficiency. Additionally, multiple laser fibers can be used together on account of their single tenuous type to improve effectiveness and adapt to a wide range.

We compared the ablative effects of LA and RFA of the myocardium in vitro. Similar results have not been reported in previous studies. However, there are several limitations in this study. First, this study used healthy porcine hearts as models that were different in terms of disease, thickness and biological structure. This may confine the use of single needle and single ablation, and the low output power was set during radiofrequency ablation. Second, this experiment only discussed the effectiveness of ablation of the myocardium in vitro, and neither live animal models nor HCOM patients were involved. As a result, some important factors were ignored, such as blood perfusion, myocardial motion and the heat sink effect. Furthermore, we did not perform pathologic studies to confirm that the ablations were complete, and the thermal field had limitations in terms of identifying incomplete ablations. Therefore, the results of this study can provide a restricted reference for animal models and HOCM patients in vivo, and more intensive exploration should be demonstrated.

## Conclusions

This study reports that the thermal ablation techniques RFA and LA are technically feasible and promising approaches for the treatment of HOCM because of their controlled and effective necrosis and the relatively secure temperature changes. We found that LA had better ablation efficiency than RFA in the ablation zone range and resulted in temperature changes with limited thermal output energy. Certainly, long-term investigations and experiments, especially in vivo assessments of animal models and HOCM patients, should be implemented.

## Data Availability

All data generated or analysed during this study are included in this published article and the datasets used and/or analysed during the current study are available from the corresponding author on reasonable request.

## Abbreviations

RFA: Radiofrequency ablation
LA: Laser ablation
HOCM: Hypertrophic obstructive cardiomyopathy
ASA: Alcohol septal ablation
RFCA: Radiofrequency catheter ablation
ICD: Implantable cardioverter defibrillator
LVOT: Left ventricular outflow tract
PIMSRA: Percutaneous intramyocardial septal radiofrequency ablation
IVS: Interventricular septum
HCC: Hepatocellular carcinoma
